# Human Safety, Tolerability, and Pharmacokinetics of Molnupiravir, a Novel Broad-Spectrum Oral Antiviral Agent with Activity against SARS-CoV-2

**DOI:** 10.1128/AAC.02428-20

**Published:** 2021-04-19

**Authors:** Wendy P. Painter, Wayne Holman, Jim A. Bush, Firas Almazedi, Hamzah Malik, Nicola C. J. E. Eraut, Merribeth J. Morin, Laura J. Szewczyk, George R. Painter

**Affiliations:** a Ridgeback Biotherapeutics LP, Miami, Florida, USA; b Covance Clinical Research Unit Limited, Leeds, United Kingdom; c Department of Pharmacology and Chemical Biology, Emory University School of Medicine, Atlanta, Georgia, USA

**Keywords:** COVID-19, EIDD-2801, SARS-CoV-2, first in human, molnupiravir, pharmacokinetics, phase 1, ribonucleoside analogue, safety, tolerability

## Abstract

Molnupiravir (EIDD-2801/MK-4482), the prodrug of the active antiviral ribonucleoside analog β-d-N4-hydroxycytidine (NHC; EIDD-1931), has activity against a number of RNA viruses, including severe acute respiratory syndrome coronavirus 2 (SARS-CoV-2), severe acute respiratory syndrome coronavirus (SARS-CoV), Middle East respiratory syndrome coronavirus (MERS-CoV), and seasonal and pandemic influenza viruses. Single and multiple doses of molnupiravir were evaluated in this first-in-human, phase 1, randomized, double-blind, placebo-controlled study in healthy volunteers, which included evaluation of the effect of food on pharmacokinetics. EIDD-1931 appeared rapidly in plasma, with a median time of maximum observed concentration of 1.00 to 1.75 h, and declined with a geometric half-life of approximately 1 h, with a slower elimination phase apparent following multiple doses or higher single doses (7.1 h at the highest dose tested). Mean maximum observed concentration (*C*_max_) and area under the plasma concentration versus time curve (AUC) increased in a dose-proportional manner, and there was no accumulation following multiple doses. When administered in a fed state, there was a decrease in the rate of absorption, but no decrease in overall exposure. Molnupiravir was well tolerated. Fewer than half of the subjects reported an adverse event, the incidence of adverse events was higher following administration of placebo, and 93.3% of adverse events were mild. One subject discontinued early due to rash. There were no serious adverse events, and there were no clinically significant findings in clinical laboratory, vital signs, or electrocardiography. Plasma exposures exceeded expected efficacious doses based on scaling from animal models; therefore, dose escalations were discontinued before a maximum tolerated dose was reached. (This study has been registered in ClinicalTrials.gov under identifier NCT04392219.)

## Abstract (precis)

Molnupiravir (EIDD-2801/MK-4482), the prodrug of the active antiviral ribonucleoside analog β-d-N4-hydroxycytidine (NHC; EIDD-1931), has activity against a number of RNA viruses, including severe acute respiratory syndrome coronavirus 2 (SARS-CoV-2), severe acute respiratory syndrome coronavirus (SARS-CoV), Middle East respiratory syndrome coronavirus (MERS-CoV), and seasonal and pandemic influenza viruses. Single and multiple doses of molnupiravir were evaluated in this first-in-human, phase 1, randomized, double-blind, placebo-controlled study in healthy volunteers, which included evaluation of the effect of food on pharmacokinetics.

## INTRODUCTION

A novel coronavirus, originally identified in Wuhan City, China, was reported to the World Health Organization on 31 December 2019 (1), and the associated disease has subsequently become a worldwide pandemic. An effective antiviral therapeutic has since been intensively sought.

**FIG 1:**
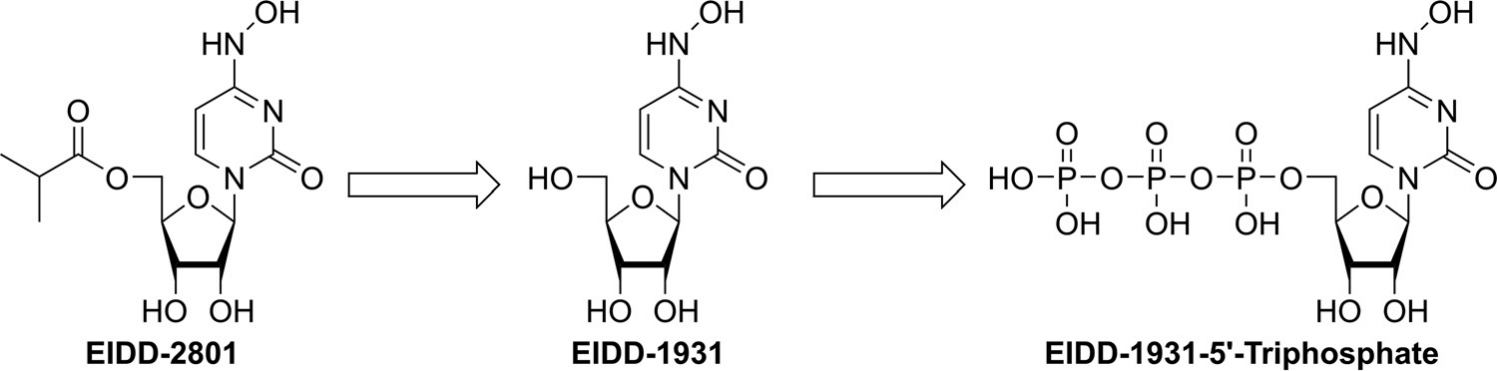
Molnupiravir is rapidly converted in the plasma to EIDD-1931 (NHC), which after distribution into various tissues is converted by host kinases into EIDD-1931 5′-triphosphate, the active antiviral agent.

Molnupiravir (also known as EIDD-2801/MK-4482) is a prodrug of the active antiviral ribonucleoside analog β-d-N4-hydroxycytidine (NHC; EIDD-1931), which has demonstrated the potential to treat infections caused by multiple RNA viruses, including highly pathogenic coronaviruses and influenza viruses, and encephalitic alphaviruses such as Venezuelan, Eastern, and Western equine encephalitis viruses, in nonclinical models (2–4). Molnupiravir is quickly cleaved in plasma to EIDD-1931, which after distribution into various tissues, is converted to its corresponding 5′-triphosphate by host kinases (Fig. 1) (4). EIDD-1931 5′-triphosphate is a competitive alternative substrate for the virally encoded RNA-dependent RNA polymerase, and upon incorporation into nascent chain viral RNA, it induces an antiviral effect via viral error catastrophe (4, 5), a concept that is predicated on increasing the viral mutation rate beyond a biologically tolerable threshold, resulting in impairment of viral fitness and leading to viral extinction. Molnupiravir has demonstrated *in vitro* activity against severe acute respiratory syndrome coronavirus 2 (SARS-CoV-2) in human airway epithelial cell cultures (2). Prophylactic and therapeutic administration of molnupiravir to mice infected with severe acute respiratory syndrome coronavirus (SARS-CoV) or Middle East respiratory syndrome coronavirus (MERS-CoV) improved pulmonary function, and reduced virus titer and body weight loss. In the ferret model of influenza, treatment of pandemic influenza A virus with molnupiravir resulted in reduced viral shedding and inflammatory cellular infiltrates in nasal lavages, with a normal humoral antiviral response (3).

Here, we report the results of a first-in-human, phase 1, randomized, double-blind, placebo-controlled study to determine the safety, tolerability, and pharmacokinetics of single and multiple ascending oral doses of molnupiravir in healthy subjects. A randomized, open-label, crossover evaluation in the fed (high fat) and fasted states was also conducted to assess the effect of food on the pharmacokinetics of single doses of molnupiravir.

## RESULTS

Eligible subjects were randomized in a 3:1 ratio to either study drug or placebo in the single- and multiple-ascending-dose parts of the study. Each cohort comprised 8 subjects, of whom 6 were administered molnupiravir and 2 were administered placebo. Single oral doses of 50 to 1,600 mg molnupiravir or placebo were administered in the single-ascending-dose part, and twice-daily (BID) doses of 50 to 800 mg molnupiravir or placebo were administered for 5.5 days in the multiple-ascending-dose part. Subjects were followed for 14 days following completion of dosing for assessments of safety, tolerability, and pharmacokinetics. Subjects in the food effect evaluation were randomized in a 1:1 ratio to either 200 mg molnupiravir (anticipated to be a therapeutic dose based on preclinical studies) in the fed state followed by 200 mg molnupiravir in the fasted state, or vice versa, with a 14-day washout period between doses. A capsule formulation was used in all parts of the study, with the exception of single ascending doses of ≤800 mg, where an oral solution formulation was used. An oral solution of the study drug in the single ascending dose part of the study allowed the greatest flexibility in initial dose escalation, based on safety and level of exposure. A capsule formulation of the study drug was used in the multiple-ascending-dose part of the study and for the food effect evaluation because this is the intended formulation for use in the clinical setting.

### Disposition.

Sixty-four subjects received a single dose of between 50 and 1,600 mg molnupiravir or placebo, 55 subjects received between 50 and 800 mg molnupiravir or placebo BID for 5.5 days, and 10 subjects received a single dose of 200 mg molnupiravir in the fed state followed by a single dose of 200 mg molnupiravir in the fasted state after a washout period of 14 days, or vice versa. Additionally, 1 subject in the multiple-ascending-dose part received 800 mg molnupiravir BID for 3 days, but was discontinued from dosing by the investigator on day 4. All subjects, including the subject who discontinued dosing, completed the protocol-specified study procedures and assessments.

### Demography.

Subjects were aged between 19 and 60 years, with a mean body mass index between 24.4 and 25.4 kg/m^2^ (Table 1). The majority of the subjects were male Caucasians. There were no other notable differences in subject demography between cohorts, except for age, where the mean age was higher in the food effect evaluation cohort, the 50-mg molnupiravir single-dose cohort, and the 100-mg molnupiravir multiple-dose cohort (data not shown).

**TABLE 1:** Study demographics

Demographic	Single dose (50–1,600 mg molnupiravir or placebo) (*N* = 64)^*c*^	Multiple dose (50–800 mg BID^*b*^ molnupiravir or placebo) (*N* = 56)	Food effect (200 mg molnupiravir) (*N* = 10)
Age (yrs)			
Mean	39.6	36.5	45.3
Range	21–60	19–60	30–60
Sex (*n*)^*a*^			
Male	53	49	7
Female	11	7	3
Race (%)			
White	95.3	92.9	90.0
Black or African-American	4.7	1.8
Other	5.4	10.0
Body mass index (kg/m^2^)			
Mean	25.05	24.44	25.38
SD	2.535	2.962	2.185

a *n*, number of observations.

b BID, twice daily.

c *N*, number of subjects.

### Tolerability.

Adverse events were graded using the Division of Microbiology and Infectious Diseases (DMID) toxicity grading (6).

### (i) Single ascending doses.

Overall, 37.5% of subjects reported an adverse event (Table 2). There were no apparent dose-related trends, with a greater proportion of subjects reporting adverse events following administration of placebo (43.8%) than following administration of molnupiravir (35.4%). Only 1 moderate adverse event (headache; grade 2) was reported following administration of molnupiravir, which occurred at the 400-mg dose level. One subject also reported moderate adverse events (nausea and headache; grade 2) following administration of placebo. No severe (grade 3) adverse events were reported. The most frequently reported adverse event was headache, which was reported by 18.8% of subjects who were administered placebo and 12.5% of subjects who were administered molnupiravir.

**TABLE 2:** Treatment-emergent adverse events (50 to 1,600 mg molnupiravir, single ascending doses)

Adverse events^*c*^	Placebo (*N* = 16)^*d*^	Molnupiravir							
		50 mg (*N* = 6)	100 mg (*N* = 6)	200 mg (*N* = 6)	400 mg (*N* = 6)	600 mg (*N* = 6)	800 mg (*N* = 6)	1,200 mg (*N* = 6)	1,600 mg (*N* = 6)
Overall (*n* [nE])	7 (12)	2 (4)	3 (4)	3 (5)	4 (7)	2 (5)	1 (2)	2 (8)
Mild (grade 1)	7 (10)	2 (4)	3 (4)	3 (4)	4 (7)	2 (5)	1 (2)	2 (8)
Moderate (grade 2)	1 (2)	1 (1)
Severe (grade 3)
Related	2 (4)	1 (1)	2 (3)	1 (1)
Preferred terms reported by more than 1 subject (*n*)^*b*^
Headache	3	1	1	3	1
Catheter site pain^*a*^	1	1	1
Nausea	1	1	1
Rhinorrhea	1	1	1
Back pain	1	1
Feeling hot	2
Pain in extremity	1	1

a Venous cannula site pain.

b Subjects who had more than 1 occurrence of the same preferred term were counted only once.

c *n*, number of subjects with an adverse event; nE, number of adverse events.

d *N*, number of subjects.

### (ii) Multiple ascending doses.

Overall, 44.6% of subjects reported an adverse event (Table 3). There were no apparent dose-related trends, with a greater proportion of subjects reporting adverse events following administration of placebo (50.0%) than following administration of molnupiravir (42.9%). With the exception of 1 subject who reported moderate (grade 2) events of oropharyngeal pain, pain in extremity, and influenza-like illness, all adverse events were mild (grade 1) in severity. The most frequently reported adverse event was diarrhea, which was reported by 7.1% of subjects who were administered molnupiravir and 7.1% of subjects who were administered placebo. One subject discontinued study drug administration on day 4 because of an adverse event of mild, truncal, maculopapular, pruritic rash following multiple BID doses of 800 mg molnupiravir, which was considered by the investigator to be related to the study drug. Following discontinuation, the subject was administered potent topical steroid treatment and antihistamines, and pruritis and rash had both resolved within 18 days.

**TABLE 3:** Treatment-emergent adverse events (50 to 800 mg molnupiravir, twice-daily multiple ascending doses)

Adverse events^*a*^	Placebo BID (*N* = 14)^*b*^	Molnupiravir BID						
		50 mg (*N* = 6)	100 mg (*N* = 6)	200 mg *N* = 6)	300 mg (*N* = 6)	400 mg (*N* = 6)	600 mg (*N* = 6)	800 mg (*N* = 6)
Overall [*n* (nE)]	7 (11)	2 (2)	3 (3)	3 (9)	2 (3)	3 (5)	2 (2)	3 (5)
Severity								
Mild (grade 1)	7 (11)	2 (2)	3 (3)	3 (6)	2 (3)	3 (5)	2 (2)	3 (5)
Moderate (grade 2)	1 (3)
Severe (grade 3)
Related	3 (4)	2 (3)	1 (1)	1 (1)	3 (4)
Preferred terms reported by more than 1 subject (*n*)^*c*^
Diarrhea	1	1	1	1
Back pain	2	1
Headache	1	2
Somnolence	2	1

a *n*, number of subjects with an adverse event; nE, number of adverse events.

b BID, twice daily; *N*, number of subjects.

c Subjects who had more than 1 occurrence of the same preferred term were counted only once.

### (iii) Food effect evaluation.

Three subjects in the food effect evaluation each reported 1 adverse event, all of which were mild (grade 1) in severity.

There were no serious adverse events reported across the entire study and there were no trends of increased frequency or severity of adverse events with higher doses of molnupiravir.

### Safety.

There were no clinically significant findings or dose-related trends in clinical laboratory, vital signs, and electrocardiogram data. There were no clinically significant changes in hematological parameters seen in this study. Dose escalations were discontinued before a maximum tolerated dose was reached because plasma exposures (as assessed by area under the plasma concentration versus time curve [AUC] and maximum observed concentration [*C*_max]_) that were expected to be efficacious based on scaling from animal models of seasonal and pandemic influenza were exceeded (3).

### Pharmacokinetics.

**(i) Single ascending doses.** Concentrations of molnupiravir were generally below the limit of quantification (BLQ) at doses up to 800 mg, with the exception of the 0.25-h time point after doses of 600 and 800 mg, where concentrations were quantifiable in 5 and 4 subjects, respectively, and the 0.5-h time point after a dose of 800 mg, where concentrations were quantifiable in all subjects. At doses of 1,200 and 1,600 mg, concentrations of molnupiravir were quantifiable at 1 or more time points between 0.25 and 1.5 h postdose in all subjects. Molnupiravir pharmacokinetic parameters were not calculable for doses of ≤400 mg; however, at doses of ≥600 mg, *C*_max_, time of *C*_max_ (*t*_max_), and time of last quantifiable concentration were reported. Following administration of between 600 and 1,600 mg molnupiravir, mean *C*_max_ values were up to 13.2 ng/ml, and values of median *t*_max_ were between 0.25 and 0.75 h (data not shown). It should be noted that molnupiravir concentrations represented only approximately 0.2% of EIDD-1931 concentrations and *t*_max_ of molnupiravir occurred at the first sampling time point for the 600-mg dose level, and therefore *C*_max_ may have been underestimated. At doses of ≥800 mg, trace amounts of molnupiravir were detected in the urine, which represented approximately 0.002% of the dose (data not shown).

**FIG 2:**
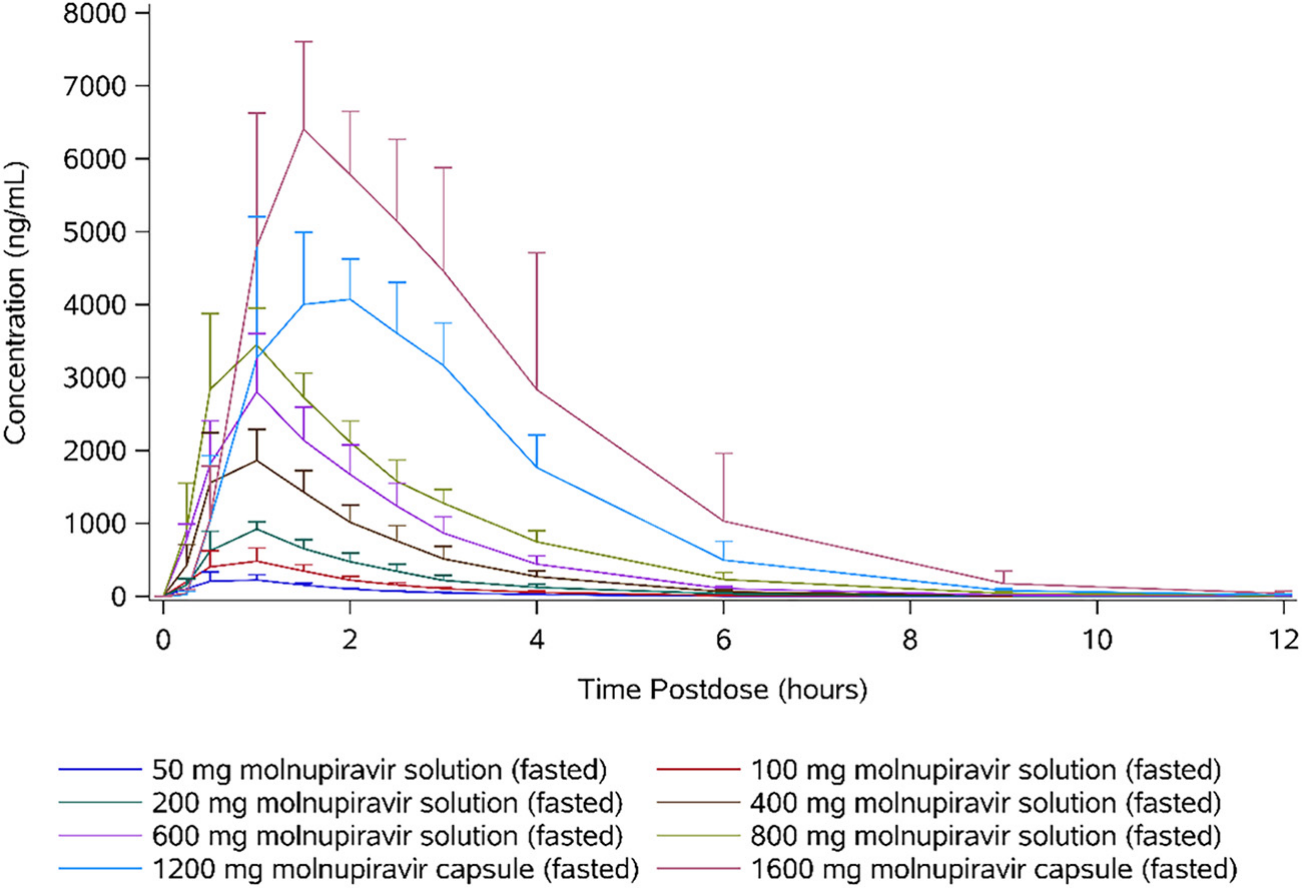
Arithmetic mean plasma concentrations of EIDD-1931 (50 to 1,600 mg molnupiravir, single ascending doses).

Following oral administration of molnupiravir at doses up to 800 mg, EIDD-1931 appeared rapidly in plasma, with a median *t*_max_ of 1.00 h postdose in all dose cohorts, after which plasma concentrations declined in an essentially monophasic manner with geometric mean terminal elimination half-lives (*t*_1/2_) of between 0.910 and 1.29 h postdose (Table 4 and Fig. 2). However, at doses of 1,200 and 1,600 mg, median *t*_max_ was delayed, with median *t*_max_ occurring at 1.75 and 1.50 h, respectively. Plasma concentrations at doses of 1,200 and 1,600 mg were quantifiable in at least 1 subject until 24 h postdose, and these profiles showed biphasic elimination with a second slower elimination phase where mean *t*_1/2_ was longer, with values of 1.81 and 4.59 h, respectively.

**TABLE 4:** Pharmacokinetic parameters of EIDD-1931 (50 to 1,600 mg molnupiravir, single ascending doses)

Parameter^*a*^	Molnupiravir^*b*^							
	50 mg (*N* = 6)	100 mg (*N* = 6)	200 mg (*N* = 6)	400 mg (*N* = 6)	600 mg (*N* = 6)	800 mg (*N* = 6)	1,200 mg (*N* = 6)	1,600 mg (*N* = 6)
AUC_last_ (ng · h/ml)	415 (27.4)	917 (27.5)	1,810 (20.0)	4,000 (20.2)	6,120 (21.6)	8,720 (10.4)	13,800 (11.7)	20,700 (31.4)
AUC_inf_ (ng · h/ml)	432 (26.5)	932 (27.0)	1,830 (19.6)	4,010 (20.2)	6,130 (21.4)	8,740 (10.4)	13,800 (11.8)	20,700 (31.4)
*C*_max_ (ng/ml)	223 (46.2)	454 (42.2)	926 (12.6)	1,850 (22.7)	2,720 (27.0)	3,640 (13.4)	4,500 (17.9)	6,350 (20.6)
*t*_max_ (h)	1.00 (0.517–1.00)	1.00 (0.500–1.50)	1.00 (0.500–1.00)	1.00 (0.500–1.00)	1.00 (1.00–1.00)	1.00 (0.500–1.00)	1.75 (1.00–2.50)	1.50 (1.00–2.00)
*t*_1/2_ (h)	0.945 (12.1)	0.907 (10.1)	1.02 (16.4)	1.03 (8.86)	1.06 (10.3)	1.29 (7.10)	1.81 (73.5)	4.59 (71.6)
Ae_0–24_ (mg)	0.323 (53.6)	1.03 (68.1)	1.51 (86.2)	5.47 (108)	14.4 (47.7)	18.0 (14.7)	53.7 (41.4)	84.4 (29.9)
Fe_0–24_ (%)	0.820 (53.6)	1.31 (68.1)	0.958 (86.2)	1.74 (108)	3.05 (47.7)	2.86 (14.7)	5.69 (41.4)	6.70 (29.9)

a Geometric means (percentage coefficient of variation) are presented, with the exception of *t*_max_, for which medians (minimum to maximum) are presented. Ae_0–24_, amount of the dose administered recovered in urine from time zero to 24 h postdose; AUC_inf_, area under the plasma concentration-time curve from time zero extrapolated to infinity; AUC_last_, area under the plasma concentration-time curve from time zero to the last measurable nonzero concentration; *C*_max_, maximum observed concentration; Fe_0–24_, percentage of the dose administered recovered in urine from time zero to 24 h postdose; *t*_1/2_, apparent terminal elimination half-life; *t*_max_, time of the maximum observed concentration.

b *N*, number of subjects;

The plasma concentration-time profiles were generally well defined, with the percentage of area under the plasma concentration-time curve from time o0extrapolated to infinity (AUC_inf_) that was extrapolated being <10% for all subjects. When assessed using a power model [ln(parameter) = intercept + slope × ln(dose) + random error], mean *C*_max_ increased in a dose-proportional manner, with the 90% confidence interval containing unity. Similarly, mean AUC_inf_ increased in an approximately dose-proportional manner; however, the lower bound of the 90% confidence interval was slightly above unity (Table 5).

**TABLE 5:** Dose proportionality of EIDD-1931 (50 to 800 mg molnupiravir, single ascending doses)

Parameter^*a*^	*n*^*b*^	Slope (90% confidence interval)	Between-subject geometric coefficient of variation	Lack of fit *P* value
AUC_inf_ (ng · h/ml)	36	1.07 (1.02–1.13)	21.5	0.9724
AUC_last_ (ng · h/ml)	36	1.09 (1.03–1.15)	21.9	0.9750
*C*_max_ (ng/ml)	36	1.01 (0.927–1.08)	29.8	0.9996

a AUC_inf_, area under the plasma concentration-time curve from time zero extrapolated to infinity; AUC_last_, area under the plasma concentration-time curve from time zero to the last measurable nonzero concentration; *C*_max_, maximum observed concentration.

b *n*, number of observations.

The amount of EIDD-1931 excreted in urine from time zero to 24 h postdose (Ae_0–24_) increased supraproportionally with dose, and there was a similar trend for apparent clearance (CL_R_) to increase. Between 0.820% (at the 50-mg dose level) and 6.70% (at the 1,600-mg dose level) of the dose was excreted in urine as EIDD-1931, and the majority of the total amount was generally excreted within the first 4 h postdose.

**(ii) Multiple ascending doses.** Concentrations of molnupiravir were generally BLQ at doses of ≤400 mg BID, and pharmacokinetic parameters were not calculable. Concentrations of molnupiravir were quantifiable in 4 subjects at either 0.5 or 1 h postdose on day 1 and in 3 subjects at 0.5 h postdose on day 6 at the 600-mg BID dose level. At the 800-mg dose level, concentrations of molnupiravir were quantifiable from all except 1 subject at 0.5 h postdose on days 1 and 6, but at no other time points, consistent with single ascending doses.

**FIG 3:**
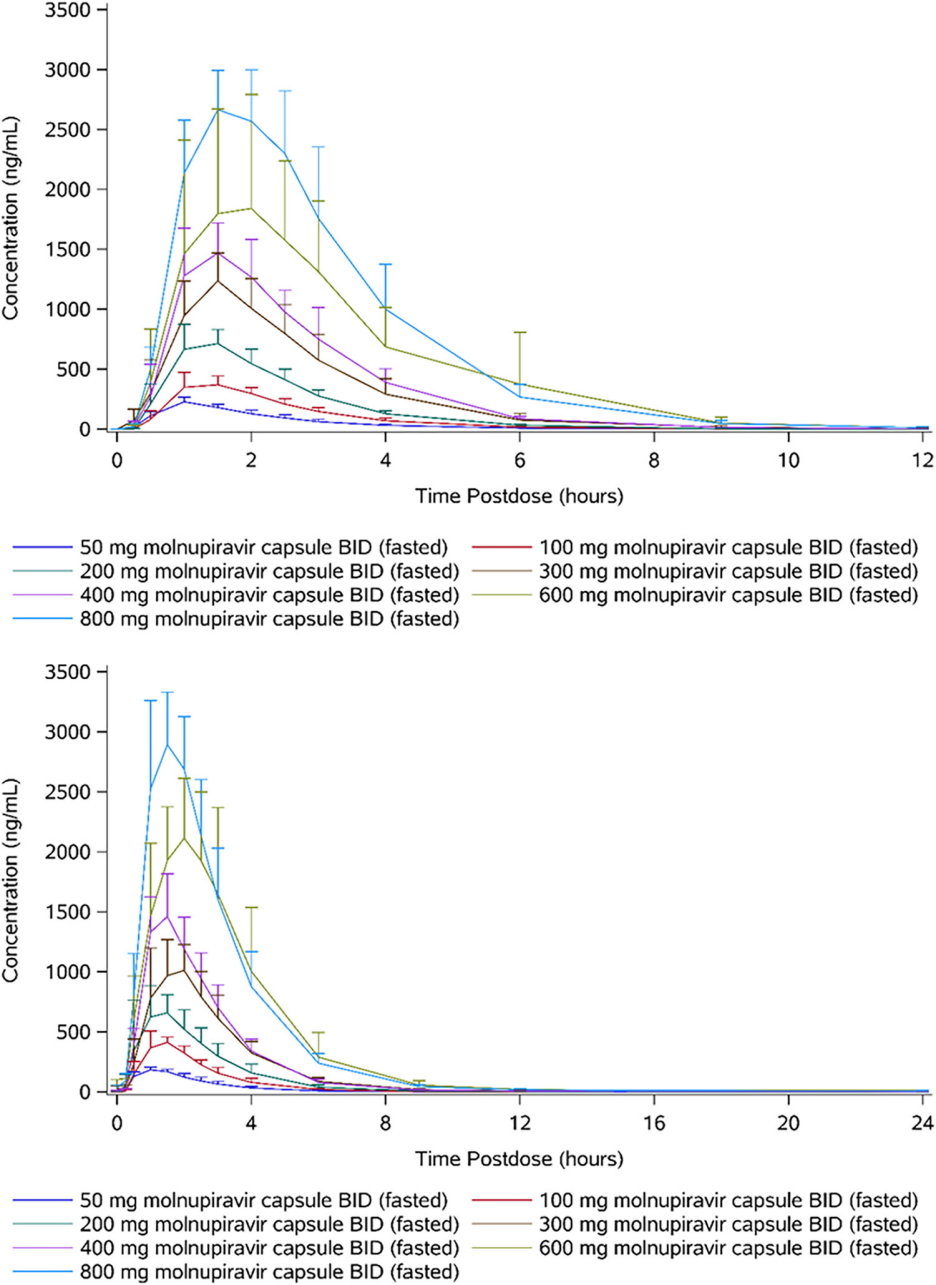
Arithmetic mean plasma concentrations of EIDD-1931 (50 to 800 mg molnupiravir, twice-daily multiple ascending doses) on day 1 (top) and day 6 (bottom).

Following oral administration of molnupiravir, EIDD-1931 appeared rapidly in plasma, with a median *t*_max_ in all dose cohorts of between 1.00 and 1.75 h postdose across both days 1 and 6 (Table 6 and Fig. 3). For all dose levels, plasma concentrations declined in an essentially monophasic manner on day 1, with mean *t*_1/2_ ranging from 0.918 to 1.18 h. Similarly, plasma concentrations declined in an essentially monophasic manner on day 6 for subjects at dose levels of ≤200 mg BID and for the majority of subjects at the 300- and 400-mg BID dose levels. In contrast, for 1 subject at each of the 300- and 400-mg dose levels and for all subjects at the 600- and 800-mg BID dose levels, biphasic elimination was observed, and there was the emergence of a second, slower elimination phase on day 6, which was reflected in an increase in the mean *t*_1/2_ with increasing dose at doses of ≥200 mg. Of note, at the 600-mg BID dose level, the lack of a clearly defined terminal elimination phase confounded the evaluation of *t*_1/2_ for the majority of subjects. At the 800-mg BID dose level, the mean *t*_1/2_ was 7.08 h.

**TABLE 6:** Pharmacokinetic parameters of EIDD-1931 (50 to 800 mg molnupiravir, twice-daily multiple ascending doses)

Parameter^*a*^	Day 1							Day 6						
	50 mg (*N* = 6)	100 mg (*N* = 6)	200 mg (*N* = 6)	300 mg (*N* = 6)	400 mg (*N* = 6)	600 mg (*N* = 6)	800 mg (*N* = 6)	50 mg (*N* = 6)	100 mg (*N* = 6)	200 mg (*N* = 6)	300 mg (*N* = 6)	400 mg (*N* = 6)	600 mg (*N* = 6)	800 mg (*N* = 5)
AUC_τ_ (ng · h/ml)	461 (15.7)	854 (19.8)	1660 (15.3)	3080 (17.3)	3800 (19.5)	6110 (26.9)	8190 (21.5)	432 (14.9)	968 (15.3)	1730 (25.2)	2960 (16.2)	3710 (21.6)	7110 (28.2)	8330 (17.9)
AUC_last_ (ng · h/ml)	444 (17.3)	835 (19.9)	1640 (15.5)	3080 (17.4)	3790 (19.5)	6110 (26.9)	8180 (21.5)	414 (16.2)	947 (15.7)	1720 (26.0)	2980 (16.3)	3730 (21.6)	7250 (28.1)	8450 (18.5)
AUC_inf_ (ng · h/ml)	461 (15.7)	855 (19.8)	1660 (15.3)	3090 (17.4)	3800 (19.5)	6680^*b*^ (17.6)	8200 (21.6)
*C*_max_ (ng/ml)	223 (19.4)	395 (18.5)	766 (16.3)	1280 (15.2)	1530 (23.2)	2160 (31.4)	2770 (13.3)	188 (8.67)	434 (14.0)	742 (32.1)	1100 (20.6)	1470 (20.9)	2240 (20.9)	2970 (16.8)
*t*_max_ (h)	1.00 (1.00–1.00)	1.25 (1.00–2.03)	1.50 (1.00–1.50)	1.50 (1.00–1.50)	1.50 (1.00–2.00)	1.75 (1.00–6.00)	1.75 (1.50–2.50)	1.00 (1.00–1.50)	1.25 (1.00–1.50)	1.50 (0.500–1.50)	1.50 (1.00–2.00)	1.50 (1.00–1.50)	1.75 (1.50–2.50)	1.50 (1.00–2.02)
MR*_C_*_max_	NC	NC	NC	NC	NC	329^*b*^ (87.7)	512 (30.6)	NC	NC	NC	NC	NC	NC	413^*c*^ (28.1)
*t*_1/2_ (h)	0.937 (14.0)	0.918 (9.08)	0.960 (10.4)	1.09 (17.7)	1.05 (13.1)	1.16^*b*^ (3.50)	1.18 (7.28)	0.968 (15.5)	0.970 (15.8)	1.24 (36.4)	1.71 (47.1)	1.20^*b*^ (9.58)	NC	7.08^*d*^ (154)
RA_AUCτ_	0.938 (7.80)	1.13 (9.25)	1.04 (18.0)	0.961 (14.7)	0.977 (11.7)	1.16 (12.2)	1.09 (11.8)
RA*_C_*_max_	0.843 (16.0)	1.10 (11.4)	0.969 (23.8)	0.861 (14.3)	0.962 (18.5)	1.04 (20.0)	1.09 (7.15)
Ae_0–12_ (mg)	0.391 (55.7)	0.993 (96.9)	1.38 (81.8)	3.37 (57.4)	4.57 (57.0)	11.9 (22.9)	22.7 (34.4)	0.336 (64.5)	0.915 (48.0)	1.76 (85.5)	3.14 (63.4)	5.32 (47.5)	16.4 (39.9)	18.9 (81.6)
Fe_0–12_ (%)	0.993 (55.7)	1.26 (96.9)	0.879 (81.8)	1.43 (57.4)	1.45 (57.0)	2.52 (22.9)	3.61 (34.4)	0.854 (64.5)	1.16 (48.0)	1.12 (85.5)	1.33 (63.4)	1.69 (47.5)	3.48 (39.9)	3.00 (81.6)

a Geometric means (percentage coefficient of variation) are presented, with the exception of *t*_max_, for which medians (minimum to maximum) are presented. Ae_0–12_, amount of dose administered recovered in urine from time zero to 12 h postdose; AUC_τ_, area under the plasma concentration-time curve during a dosing interval; AUC_inf_, area under the plasma concentration-time curve from time zero extrapolated to infinity; AUC_last_, area under the plasma concentration-time curve from time zero to the last measurable nonzero concentration; *C*_max_, maximum observed concentration; Fe_0–12_, percentage of the dose administered recovered in urine from time zero to 12 h postdose; MR, metabolite ratio; N, number of subjects; NC, not calculated due to insufficient molnupiravir (prodrug) concentrations above the lower limit of quantification; RA, accumulation ratio; *t*_1/2_, apparent terminal elimination half-life; *t*_max_, time of the maximum observed concentration.

b *N* = 5.

c *N* = 3.

d *N* = 4.

There was no evidence of accumulation, with the geometric mean accumulation ratios based on area under the plasma concentration-time curve during a dosing interval (AUC_τ_) and *C*_max_ values between 0.938 and 1.16 and between 0.843 and 1.10, respectively, across all dose levels.

On day 1, when assessed using the power model, mean *C*_max_ and AUC_inf_ increased in an approximately dose-proportional manner. However, the upper bound of the 90% confidence interval for *C*_max_ was slightly below unity and the lower bound of the 90% confidence interval for AUC_inf_ was slightly above unity (Table 7). On day 6, mean *C*_max_ increased in a dose-proportional manner, with the 90% confidence interval containing unity. Similarly, mean AUC_τ_ increased in an approximately dose-proportional manner; however, the lower bound of the 90% confidence interval was slightly above unity (Table 7).

**TABLE 7:** Dose proportionality of EIDD-1931^*a*^

Parameter^*b*^	Day 1				Day 6			
	*n*^*c*^	Slope (90% confidence interval)	Between-subject geometric coefficient of variation	Lack of fit *P* value	*n*	Slope (90% confidence interval)	Between-subject geometric coefficient of variation	Lack of fit *P* value
AUC_inf_ (ng · h/ml)	53	1.10 (1.06, 1.14)	19.6	0.3149
AUC_last_ (ng · h/ml)	54	1.11 (1.07, 1.15)	20.8	0.4483
AUC_τ_ (ng · h/ml)	41	1.08 (1.02–1.14)	20.5	0.3670
*C*_max_ (ng/ml)	54	0.957 (0.916, 0.998)	20.1	0.7011	41	0.971 (0.915–1.03)	20.3	0.7798

a 50 to 1,600 mg molnupiravir, single ascending doses (day 1), and 50 to 800 mg molnupiravir, twice-daily multiple ascending doses (day 6).

b AUC_τ_, area under the plasma concentration-time curve during a dosing interval; AUC_inf_, area under the plasma concentration-time curve from time zero extrapolated to infinity; AUC_last_, area under the plasma concentration-time curve from time zero to the last measurable nonzero concentration; *C*_max_, maximum observed concentration.

c *n*, number of observations.

AUC_inf_ on day 1 for the multiple-dose cohorts, where a capsule formulation was administered, was similar to those for the corresponding single-dose cohorts where a solution formulation was administered, with geometric mean ratios of between 0.91 and 1.09. Geometric mean *C*_max_ was slightly lower following dosing with the capsule formulation, with geometric mean ratios of between 0.76 and 1.00, and a trend to smaller ratios at higher doses. Median *t*_max_ occurred up to 0.75 h later following administration of the capsule formulation, with the difference being greatest at doses ≥600 mg BID. Thus, it appears that the extent of absorption is similar for the solution and capsule formulations, but the rate of absorption appears to be slightly slower for the capsules. However, these data should be interpreted with caution because this was not a crossover study.

Between 0.854% and 3.61% of the dose was excreted in urine as EIDD-1931 on both days 1 and 6, and, similar to single doses, the majority was excreted in the first 4 h postdose (Table 6). There was no consistent dose-related trend in the percentage of dose administered recovered in urine during a dosing interval (Fe_0–τ_) or CL_R_ at doses of ≤200 mg BID. However, there was a trend for Fe_0–τ_ and CL_R_ to increase with dose at doses >200 mg BID, with a 4-fold increase in dose from 200 to 800 mg BID resulting in a 16-fold increase in the amount of the dose recovered in urine during a dosing interval (Ae_0–τ_) on day 1 and an 11-fold increase in Ae_0–τ_ on day 6.

**(iii) Food effect.** Concentrations of molnupiravir were generally BLQ and pharmacokinetic parameters were not calculable. Concentrations of EIDD-1931 were quantifiable at 0.25 h postdose for 2 subjects in the fasted state, but no subjects in the fed state. The first quantifiable concentrations in the fed state were between 0.5 and 1.5 h postdose.

**FIG 4:**
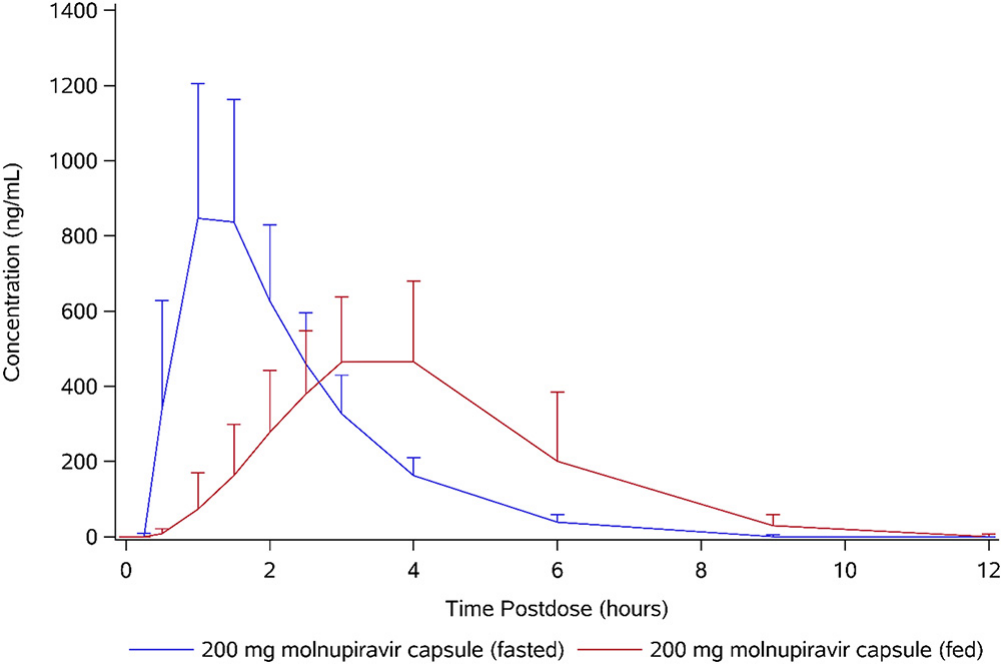
Arithmetic mean plasma concentration of EIDD-1931 (food effect).

Following administration of 200 mg molnupiravir in the fed state, *t*_max_ of EIDD-1931 occurred later, with a median of 3.00 h postdose versus 1.00 h postdose (Table 8 and Fig. 4). Generally, the slower absorption and later *t*_max_ in the fed state was reflected in lower *C*_max_; however, one subject had similar profiles for both treatments (data not shown). Mean *C*_max_ was approximately 36% lower in the fed state compared to the fasted state, and this difference was statistically significant; however, exposure (assessed by AUC_inf_) was similar for both fed and fasted states and demonstrated that the rate, but not the extent, of absorption was lower in the fed state. Following *C*_max_, concentrations of EIDD-1931 declined in an essentially monophasic manner in both the fed and fasted state and remained quantifiable until between 9 and 15 h postdose in the fed state and between 6 and 9 h in the fasted state. The mean *t*_1/2_ was similar between fed and fasted treatments, with values of 1.09 and 0.977 h, respectively. Urine pharmacokinetic parameters were similar to those reported for single ascending doses.

**TABLE 8:** Pharmacokinetic parameters of EIDD-1931 (food effect)

Parameter^*a*^	200 mg EIDD-1931	
	Fasted) (*N* = 10)	Fed (*N* = 10)
AUC_last_ (ng · h/ml)	1,950 (29.9)	1,870 (29.0)
AUC_inf_ (ng · h/ml)	1,980 (29.3)	1,890 (28.8)
*C*_max_ (ng/ml)	893 (36.8)	575 (27.7)
*t*_max_ (h)	1.00 (1.00–2.50)	3.00 (2.00–4.00)
*t*_1/2_ (h)	0.977 (13.0)	1.09 (16.3)
FE_AUCinf_	0.955 (9.7)
FE*_C_*_max_	0.644 (22.5)
Ae_0–24_ (mg)	1.76 (45.6)	1.63 (54.2)
Fe_0–24_ (%)	1.12 (45.6)	1.04 (54.2)

a Geometric means (percentage coefficient of variation) are presented, with the exception of *t*_max_, for which medians (minimum to maximum) are presented. Ae_0–24_, amount of the dose administered recovered in urine from time zero to 24 h postdose; AUC_inf_, area under the plasma concentration-time curve from time zero extrapolated to infinity; AUC_last_, area under the plasma concentration-time curve from time zero to the last measurable nonzero concentration; *C*_max_, maximum observed concentration; Fe_0–24_, percentage of the dose administered recovered in urine from time zero to 24 h postdose; FE_AUCinf_, ratio of area under the plasma concentration-time curve from time zero extrapolated to infinity (fed:fasted); FE*_C_*_max_, ratio of maximum observed concentration (fed:fasted); *t*_1/2_, apparent terminal elimination half-life; *t*_max_, time of the maximum observed concentration.

## DISCUSSION

There is a significant need for an antiviral drug against coronaviruses with pandemic potential that is generally safe and well tolerated and can be easily administered in the outpatient setting. The oral route of administration of molnupiravir makes it appropriate and convenient for administration to outpatients.

Molnupiravir is well absorbed and the appearance of the parent ribonucleoside analog, EIDD-1931, in plasma demonstrates linear, dose-proportional pharmacokinetics when administered between doses of 50 and 1,600 mg. Although the rate of absorption was slower in the fed state, with lower values of *t*_max_ and *C*_max_ and a longer duration of measurable exposure, the extent of absorption (as assessed by AUC_inf_) was similar for both fed and fasted states. Therefore, the administration of molnupiravir with food is unlikely to have an effect on therapeutic exposure. There was no evidence in humans of the capacity-limited uptake observed in pharmacokinetic studies conducted in mice (4). Exposures in the multiple-ascending dose part of the study, which utilized doses between 50 and 800 mg administered BID, could be extrapolated from the exposures observed in the single-ascending dose part of the study. The *t*_1/2_ of EIDD-1931 is dose dependent, and ranges between 0.907 and 7.08 h. However, it should be noted that the decision to utilize BID dosing in the multiple ascending dose part of the study was based on *t*_1/2_ values for the active antiviral agent, EIDD-1931 5′-triphosphate, determined in cell culture and in lung tissue from animal model studies (4, 7). In these studies, the intracellular half-life of EIDD-1931 5′-triphosphate ranged from 3 h in Huh-7 cells, a human liver cell line, up to 6.6 h in murine lung tissue.

Molnupiravir was well tolerated at doses of 50 to 800 mg administered BID for 5.5 days and at single doses up to 1,600 mg. The most frequently observed adverse event was headache in the single-ascending dose part and diarrhea in the multiple-ascending dose part. A greater number of placebo-treated subjects reported headaches in the single-ascending dose part (18.8% placebo versus 12.5% molnupiravir) and the same number of placebo-treated subjects reported diarrhea in the multiple-ascending dose part (7.1%) as molnupiravir-administered subjects. One subject discontinued dosing in this study because of rash. No subjects experienced serious adverse events. The absence of clinically significant findings or dose-related trends in clinical laboratory, vital signs, and electrocardiography, taken with the tolerability findings, indicate that molnupiravir was generally safe at the dose levels and duration tested in this study cohort. Homogeneity in body mass index and other enrollment restrictions that minimize metabolic differences among subjects (for example, diabetes and gastrointestinal disease, etc.) may have reduced variability in pharmacokinetic parameters. While most participants were male and Caucasian because of enrollment criteria and geographic constraints of the study site, there are no known additional metabolism concerns that are sex- or race-based. Data from this study support advancement of molnupiravir into phase 2 studies in subjects with susceptible RNA-viral diseases, including COVID-19.

The ability of molnupiravir to potentially treat highly pathogenic respiratory RNA virus infections has been demonstrated in ferret models of disease. In ferrets, molnupiravir was highly effective at treating seasonal and pandemic influenza infections and in blocking SARS-CoV-2 transmission. The lowest fully efficacious doses were 2.3 mg/kg of body weight against seasonal influenza and 7 mg/kg of body weight against pandemic influenza (3). A therapeutic dose of 5 mg/kg was completely effective in blocking SARS-CoV-2 transmission from infected animals that shed virus to cohoused, uninfected animals (8). The doses that were effective in ferrets yielded plasma exposure levels ranging between 466 and 1,418 ng/ml for *C*_max_ and between 1,474 and 4,487 ng · h/ml for AUC. Based on both *C*_max_ and AUC levels achieved in the phase 1 study (Table 4), adequate plasma exposure to be effective in treating seasonal and pandemic influenza and in blocking the transmission of SARS-CoV-2 in animal models is reached in humans at doses between 200 and 800 mg.

Molnupiravir was also highly effective when administered prophylactically and therapeutically in mouse models of SARS-CoV-2 and MERS-CoV. Doses of 50, 150, and 500 mg/kg BID were effective in significantly reducing lung viral loads and in improving pulmonary function (2). Scaling between efficacious molnupiravir exposures in mouse models of coronavirus infection and in humans is significantly more difficult than in ferrets. High plasma levels of EIDD-1931 are required to achieve efficacious exposures to EIDD-1931 5′-triphosphate in murine lung tissue (4) because of two key factors. First, mice have between 45% and 94% higher plasma uridine levels than humans (9–11). Uptake of EIDD-1931 from the plasma into tissue appears to be mediated by sodium-dependent concentrative transporters that show a high affinity for uridine. High plasma levels of uridine can effectively outcompete the uptake and distribution of EIDD-1931 from the plasma into key tissues in the pathogenesis of disease. Second, levels of uridine in mouse tissues are often 10-fold higher than the levels in plasma (9). Consequently, uridine can also compete with EIDD-1931 for phosphorylation. Therefore, the levels of uridine seen in mouse plasma and tissue can result in significantly higher concentrations of drug being required to produce efficacious levels of active 5′-triphosphate at the site of drug action. Nonetheless, exposures (based on AUC levels) that were associated with therapeutic effects in mice were achieved in humans.

In conclusion, molnupiravir is well absorbed after oral administration and absorption is minimally affected by food intake. Plasma exposure to EIDD-1931 is essentially dose proportional across the range of doses tested, and based on scaling from animal models of influenza and pathogenic coronavirus infections, these plasma exposures may be adequate to provide prophylactic and therapeutic benefit in the treatment of these respiratory infections. No accumulation was observed in the multiple-ascending-dose part of this study. Very little molnupiravir or EIDD-1931 was detected in urine, despite the fact that nucleoside analogs as well as natural nucleosides are in general actively secreted by the kidney. This may be the result of metabolism of EIDD-1931 to cytidine and uridine. Molnupiravir was well tolerated in this study, and no subjects experienced serious adverse events.

Molnupiravir, having demonstrated good tolerability and dose-proportional pharmacokinetics following administration to healthy volunteers at clinically relevant doses is well positioned to be evaluated for clinical efficacy and safety in large-scale COVID-19 studies.

## MATERIALS AND METHODS

### Study population.

Healthy subjects, of any ethnic origin, race, or sex, aged between 18 and 60 years (inclusive), and with a body mass index between 18 and 30 kg/m^2^ (inclusive), were eligible to participate in this study. Potential subjects were screened for inclusion in the study within 28 days prior to the first dose administration after voluntarily providing informed consent. Subjects were not permitted to participate in more than 1 part of the study or to be enrolled into more than 1 cohort.

### Study design.

This study was performed at a single clinical research unit in the United Kingdom. Eligible subjects were randomized in a 3:1 ratio to either study drug or placebo in the single- and multiple-ascending-dose parts using a computer-generated pseudorandom procedure at the time of first dose administration. Placebo was chosen as the control treatment to assess whether any observed safety and tolerability effects were related to the treatment or simply reflected the study conditions. Each dose escalation cohort comprised 8 subjects, of whom 6 were administered molnupiravir and 2 were administered placebo. Single oral doses of 50 to 1,600 mg molnupiravir or placebo were administered in the single-ascending-dose part and BID doses of between 50 and 800 mg molnupiravir or placebo were administered for 5 days with a final dose on the morning of day 6 in the multiple-ascending-dose part. Safety and tolerability data up to 72 h postdose (post final dose for multiple ascending doses) were reviewed prior to each dose escalation to ensure that it was safe to proceed with the planned design, and multiple doses were not administered until the planned total daily dose had been shown to be safe and well tolerated as a single dose. Subjects were followed for 14 days following dose administration (post final dose for subjects who received multiple doses) for assessments of safety, tolerability, and pharmacokinetics, which exceeded 5 half-lives of EIDD-1931 observed in nonclinical studies (*t*_1/2_ = 9.1 h in dogs and 5 h in ferrets).

Subjects in the food effect evaluation were randomized in a 1:1 ratio to either 200 mg molnupiravir in the fed state followed by 200 mg molnupiravir in the fasted state, or vice versa. Randomization was performed and subjects were followed as described for the single- and multiple-ascending-dose parts. There was a 14-day washout period between doses in the food effect evaluation.

This study was of an adaptive design. The plasma and urine pharmacokinetic sampling time points for the single- and multiple-ascending dose parts and the food-effect evaluation are presented in Table S1 (plasma) and Table S2 (urine) in the supplemental material.

A capsule formulation was used in all parts of the study, with the exception of single ascending doses of ≤800 mg, where an oral solution formulation was used.

The study was conducted in accordance with the International Council for Harmonisation Good Clinical Practice guidelines, the ethical principles outlined in the Declaration of Helsinki, and European Union Clinical Trial Directive 2001/20/EC, following approval by applicable regulatory authorities and receipt of a favorable opinion by an ethics committee.

### Objectives and endpoints.

The primary objectives of this study were (i) to determine the safety and tolerability of single and multiple ascending doses of molnupiravir, and (ii) to assess the effect of food on the pharmacokinetics of molnupiravir and EIDD-1931 following a single dose. The secondary objective of the study was to define the pharmacokinetics of molnupiravir and EIDD-1931 in plasma and urine following single and multiple doses in healthy subjects. The safety and tolerability endpoints were clinical laboratory evaluations, vital signs, electrocardiograms, physical examinations, and adverse events. Pharmacokinetic endpoints included plasma and urinary parameters.

### Criteria for evaluation.

Following single doses, plasma and urine samples for pharmacokinetic analysis were collected up to 72 and 48 h postdose, respectively. Following multiple doses, plasma and urine samples were collected up to 12 h after the first dose and up to 192 and 72 h postdose, respectively, after the final dose. Pharmacokinetic samples were analyzed for molnupiravir and EIDD-1931 using a validated liquid chromatography with tandem mass spectrometry bioanalytical method. The validated ranges were 5 to 5,000 ng/ml in plasma and 25 to 25,000 ng/ml in urine. The accuracy of the bioanalytical method was determined using incurred sample reanalysis, during which it was observed that all results had a relative percentage difference within the acceptable criteria of ±20%. Pharmacokinetic parameters were calculated using noncompartmental methods in Phoenix WinNonlin version 8.1.

Plasma concentrations that were BLQ were set to 0, with the exceptions of (i) any BLQ concentration that was embedded between 2 quantifiable concentrations, (ii) any BLQ concentrations that occurred after the last quantifiable concentration, or (iii) where an entire concentration-time profile was BLQ; any concentrations that met any of these criteria were set to “missing.” Urine concentrations that were BLQ were set to 0 for the calculation of the amount excreted in urine.

Safety and tolerability were monitored throughout study participation through recording of adverse events, clinical laboratory evaluations, vital signs, electrocardiogram measurements, and physical examinations. Adverse events were monitored by observation of signs and symptoms, open questioning, and spontaneous reporting, and were graded according to the National Institutes of Health Division of Microbiology and Infectious Disease toxicity grading scale. Subjects who had more than 1 occurrence of the same adverse event were counted only once.

### Sample size and statistical analysis.

No formal sample size calculation was conducted; however, 8 subjects per cohort was considered sufficient for an adequate pharmacokinetic analysis following single and multiple doses. Ten subjects in the food effect evaluation was typical of food effect studies with a randomized crossover design. Data were presented using descriptive statistics.

### Data availability.

Certain data (for example, subject-level data) will be withheld from disclosure due to national and/or regional regulatory requirements and data protection policies. Other data will be available on request within 6 months of this publication via direct communication with the corresponding author.

## Supplementary Material

Supplemental file 1
