# Supplementary material for: Human Safety, Tolerability, and Pharmacokinetics of Molnupiravir, a Novel Broad-Spectrum Oral Antiviral Agent with Activity against SARS-CoV-2
Source: Antimicrob Agents Chemother. 2021 Apr 19;65(5):e02428-20. doi: 10.1128/AAC.02428-20 (PMC8092915; doi:10.1128/AAC.02428-20)
Supplement: Supplemental file 1 [file AAC.02428-20-s0001.pdf]

Human Safety, Tolerability, and Pharmacokinetics of Molnupiravir, a Novel Broad-Spectrum Oral Antiviral Agent with Activity Against SARS-CoV-2

Wendy P. Painter, Wayne Holman, Jim A. Bush, Firas Almazedi, Hamzah Malik, Nicola C. J. E. Eraut, Merribeth J. Morin, Laura J. Szewczyk, George R. Painter

Supplementary Material

Table S1 Plasma Pharmacokinetic Sampling Timepoints

Single Ascending Doses

		Postdose Timepoints (Hours)															
**Molnupiravir Dose**	**Predose**	**0.25**	**0.5**	**1**	**1.5**	**2**	**2.5**	**3**	**4**	**6**	**9**	**12**	**15**	**24**	**36**	**48**	**72**
50 mg	X		X	X	X	X	X	X	X	X	X	X	X	X	X	X	X
100 mg	X		X	X	X	X	X	X	X	X	X	X	X	X	X	X	X
200 mg	X	X	X	X	X	X	X	X	X	X	X	X	X	X	X	X	X
400 mg	X	X	X	X	X	X	X	X	X	X	X	X		X			
600 mg	X	X	X	X	X	X	X	X	X	X	X	X		X			
800 mg	X	X	X	X	X	X	X	X	X	X	X	X		X			
1200 mg	X	X	X	X	X	X	X	X	X	X	X	X		X			
1600 mg	X	X	X	X	X	X	X	X	X	X	X	X	X	X			

Food-Effect

		Postdose Timepoints (Hours)															
**Molnupiravir Dose**	**Predose**	**0.25**	**0.5**	**1**	**1.5**	**2**	**2.5**	**3**	**4**	**6**	**9**	**12**	**15**	**24**	**36***	**48***	**72***
200 mg	X	X	X	X	X	X	X	X	X	X	X	X	X	X	X	X	X

* Samples only collected in the first treatment period.

Multiple Ascending Doses

		Postdose Timepoints (Hours)*																
**Molnupiravir Dose**	**Predose**	**0.25**	**0.5**	**1**	**1.5**	**2**	**2.5**	**3**	**4**	**6**	**9**	**12**	**15†**	**24†**	**36†**	**48†**	**72†**	**192†**	**Day 4 Predose**
50 mg	X	X	X	X	X	X	X	X	X	X	X	X	X	X		X	X	X	X
100 mg	X	X	X	X	X	X	X	X	X	X	X	X		X					X
200 mg	X	X	X	X	X	X	X	X	X	X	X	X		X					X
300 mg	X	X	X	X	X	X	X	X	X	X	X	X		X					X
400 mg	X	X	X	X	X	X	X	X	X	X	X	X		X					X
600 mg	X	X	X	X	X	X	X	X	X	X	X	X	X	X					X
800 mg	X	X	X	X	X	X	X	X	X	X	X	X	X	X	X				X

* Timepoints relative to dose administration on Days 1 and 6.

† Sample collection following dose administration on Day 6 only.

Table S2 Urine Pharmacokinetic Sampling Timepoints

Single Ascending Doses

		Postdose Time Windows (Hours)					
**Molnupiravir Dose**	**Predose**	**0 to 4**	**4 to 8**	**8 to 12**	**12 to 24**	**24 to 36**	**36 to 48**
50 mg	X	X	X	X	X	X	X
100 mg	X	X	X	X	X	X	X
200 mg	X	X	X	X	X	X	X
400 mg	X	X	X	X	X		
600 mg	X	X	X	X	X		
800 mg	X	X	X	X	X		
1200 mg	X	X	X	X	X		
1600 mg	X	X	X	X	X		

Food-Effect

		Postdose Time Windows (Hours)					
**Molnupiravir Dose**	**Predose**	**0 to 4**	**4 to 8**	**8 to 12**	**12 to 24**	**24 to 36***	**36 to 48***
200 mg	X	X	X	X	X	X	X

* Samples only collected in the first treatment period.

Multiple Ascending Doses

		Postdose Time Windows (Hours)*					
**Molnupiravir Dose**	**Predose**	**0 to 4**	**4 to 8**	**8 to 12**	**12 to 24†**	**24 to 48†**	**48 to 72†**
50 mg	X	X	X	X	X	X	X
100 mg	X	X	X	X	X		
200 mg	X	X	X	X	X		
300 mg	X	X	X	X	X		
400 mg	X	X	X	X	X		
600 mg	X	X	X	X	X		
800 mg	X	X	X	X	X		

* Time windows relative to dose administration on Days 1 and 6.
† Sample collection following dose administration on Day 6 only.
